# Different Visual Preference Patterns in Response to Simple and Complex Dynamic Social Stimuli in Preschool-Aged Children with Autism Spectrum Disorders

**DOI:** 10.1371/journal.pone.0122280

**Published:** 2015-03-17

**Authors:** Lijuan Shi, Yuanyue Zhou, Jianjun Ou, Jingbo Gong, Suhong Wang, Xilong Cui, Hailong Lyu, Jingping Zhao, Xuerong Luo

**Affiliations:** 1 Mental Health Institute of The Second Xiangya Hospital and Key Laboratory of Psychiatry and Mental Health of Hunan Province, The Central South University, Changsha, Hunan, P.R. China; 2 Hangzhou Seventh People’s Hospital, Hangzhou, Zhejiang, P.R. China; 3 Traditional Chinese Medicine University of Hunan, Changsha, Hunan, P.R. China; 4 Department of Neuroscience, The Third Affiliated Hospital of Soochow University, Changzhou, Jiangsu, P.R. China; University of Udine, ITALY

## Abstract

Eye-tracking studies in young children with autism spectrum disorder (ASD) have shown a visual attention preference for geometric patterns when viewing paired dynamic social images (DSIs) and dynamic geometric images (DGIs). In the present study, eye-tracking of two different paired presentations of DSIs and DGIs was monitored in a group of 13 children aged 4 to 6 years with ASD and 20 chronologically age-matched typically developing children (TDC). The results indicated that compared with the control group, children with ASD attended significantly less to DSIs showing two or more children playing than to similar DSIs showing a single child. Visual attention preference in 4- to 6-year-old children with ASDs, therefore, appears to be modulated by the type of visual stimuli.

## Introduction

Autism spectrum disorders (ASDs) are neurodevelopmental disorders marked by impairments in social interaction and communication, as well as repetitive and restricted behaviors[1]. Among these symptoms, impairments in social interaction are considered the most basic characteristic, and are thought to be partially due to abnormal perception of social information[2]. As observed in most studies, the visual preference for social stimuli present in typically developed populations are not present in ASD, while a range of physical stimuli (such as rotating vehicle wheels, particular sounds) appear to be preferential for the ASD population[3]. Several recent studies using modern infrared eye-tracking technology have provided new insights into the visual attention patterns associated with ASDs, and have quantified many abnormalities in visual attention preferences for social information in the ASD population[4–11].

Using a paired visual comparison procedure in which subjects are presented simultaneously with images of social content (e.g., a human face) and non-social content related to a circumscribed interest (e.g., trains), Sasson and Touchstone [7] reported that autistic children presented a preference for objects related to the circumscribed interest. Pierce and colleagues[9] demonstrated that young children with ASD spent significantly more time fixating on dynamic geometric images (DGIs) than did age-matched children with delayed and typical development characteristics. Furthermore, they reported that, if a toddler spent more than 69% of his or her time fixating on geometric patterns, the positive predictive value of accurately classifying that toddler as having an ASD was 100%. The findings of this study are encouraging for the development of eye-tracking-based early identification models of ASD.

However, some studies have reported no difference in the visual attention paid to dynamic visual stimulation objects (e.g., faces) between children with ASD and typically developing children (TDC), and found that all participants were attracted to high-salience objects[12]. The heterogeneity in sample age may be an important factor responsible for this discrepancy. The participants in the study by Pierce et al. were infants as young as 14 months, while Parish-Morris et al. studied older children in the age range from 6 to 17 years. Some studies have indicated that these visual attention patterns change over time and the gaze pattern differs between children and adults with ASD[4,13]. The difference in experimental stimulus may also contribute to this discrepancy. Chawarska and colleagues[14] found that in certain contexts, for example, when there is only one person in the visual field, young children with autism exhibit the same visual attention preference patterns in response to people and objects as TDC; only when more complex cues were introduced did the differences between the autism and control groups become apparent.

The present study was conducted to investigate the impact of age and stimulus types on visual social attention in children with ASD. In this study, we recruited children aged 4 to 6 years because it is difficult to diagnose ASD in younger children. The average age at diagnosis was 3.1 years for children with autism, and 3.9 years for pervasive developmental disorders not otherwise specified [15,16]. The aim of this study was to compare the differences in visual social attention patterns between children with ASD and TDC. To achieve this, it is essential that the diagnosis of ASD is clear. Pierce et al. reported the visual social attention patterns for children aged 2 to 3 years; therefore, in the present study, we explored the visual social attention patterns of older children. In Part I of the study, two groups of children aged 4 to 6 years (the ASD and TDC groups) were evaluated using the stimuli reported by Pierce et al[9]. In Part II, the same participants were evaluated using modified stimuli in which DSIs contained two or more children playing and DGIs comprised images of letters and geometric shapes. Based on previous research, it was hypothesized that the findings of Pierce et al.[9] are not representative of preschool-aged children with ASD, and that more complex social engagement can elicit differences between groups of children with ASD and TDC.

## Methods

### Ethics Statement

The research protocol for this study was approved by the Ethics Committee of the Second Xiangya Hospital, Central South University, China. Written informed consent was obtained from the parents or legal guardians before the participants were enrolled. The individual in this manuscript has given written informed consent (as outlined in PLOS consent form) to publish these case details.

### Participants

Children in the ASD group were recruited from special education schools or outpatients of the Second Xiangya Hospital. Inclusion criteria were: a previous DSM-IV diagnosis of an ASD made by a licensed clinician experienced in the assessment and diagnosis of autism and a diagnosis confirmation made using the Autism Diagnostic Observation Schedule [17] and the Autism Diagnostic Interview-Revised [18]. TDC were recruited from a kindergarten in the same city. TDC who were reported to have any developmental disorder (e.g. mental retardation, or learning disability) or psychiatric disorder, or any immediate family members with an ASD diagnosis were excluded. For both groups, none of the participants had a history of seizure-associated disorders, acute medical or genetic conditions, or any visual impairment that was uncorrectable with prescription lenses. The two groups were matched in terms of chronological age, not developmental age, to reduce any influence of age-related deviation in object preference. The verbal intelligence quotients of all participants were assessed using the Chinese version of the Peabody Picture Vocabulary Test (PPVT)[19].

Forty three preschool children participated in this study: 20 with ASD and 23 TDC. In Part I, two children with ASD were excluded from the analyses because their viewing time on the screen was less than 50% of the total time (i.e., 30 s). In Part II, seven children with ASD and three TDC were excluded for poor compliance during eye-tracking tasks. The final sample of 33 children who completed both parts of the experiment consisted of 13 with ASD (12 males, 1 female; mean age = 60.85 months, range = 37–98 months) and 20 TDC (14 males, 6 females; mean age = 58.10 months, range = 38–91 months). There was no significant difference in age (t (31) = -0.520, *p* = 0.607) and sex ratio (*p* = 0.202) between the ASD and TDC groups, but PPVT VIQ did differ between the two groups (t (31) = 12.581, *p* < 0.001). Further demographic and examination data are shown in Table 1.

**Table 1:** Sample characteristics.

Characteristics	ASD Mean (SD)(range)	TDC Mean(SD)	*t*-value	*p*-value
**Sex (M/F)**	12/1	14/6		0.202
**Age, months,**	60.85(16.29)	58.10(13.80)	−0.520	0.607
**PPVT VIQ**	55.77(10.22)	116.10(15.16)	12.581	<0.001
**ADOS social affect score**	15.08(2.72)(10–21)			
**ADOS restricted repetitive score**	2.15(1.35)(0–4)			
**ADOS total score**	17.23(3.61)(10–24)			
**ADI-R A total scores**	18.46(5.14)(10–24)			
**ADI-R B total scores**	15.31(4.39)(9–21)			
**ADI-R C total scores**	2.92(2.06)(0–7)			
**ADI-R D total scores**	4.08(1.32)(1–5)			

ASD: autism spectrum disorder; TDC: typically developing children; PPVT VIQ: Peabody Picture Vocabulary Test Verbal Intelligence Quotient; ADOS: Autism Diagnostic Observation Schedule; ADI-R: Autism Diagnostic Interview-Revised; the sex composition of the two groups was tested using the Fisher exact test.

### Apparatus

Infrared eye-tracking recording was performed using EyeLink1000 (SR Research, Ontario, Canada; 500 Hz) with a remote camera. The device had a tolerance for head motion of 22 cm horizontally, 18 cm vertically and 20 cm in depth. The monocular eye-tracker used infrared light sources and cameras that are integrated into a 60 cm display viewed from a distance of 70 cm and a subtended 41.4 h × 23.9 v degree visual angle.

### Stimuli

The stimuli used in the original design reported by Pierce et al. were used in Part I of this study. These stimuli comprised a movie consisting of DGIs (recordings of animated screen saver programs) on one half of the screen and DSIs (a series of short sequences of children doing yoga) on the other half [9].

**Fig 1:**
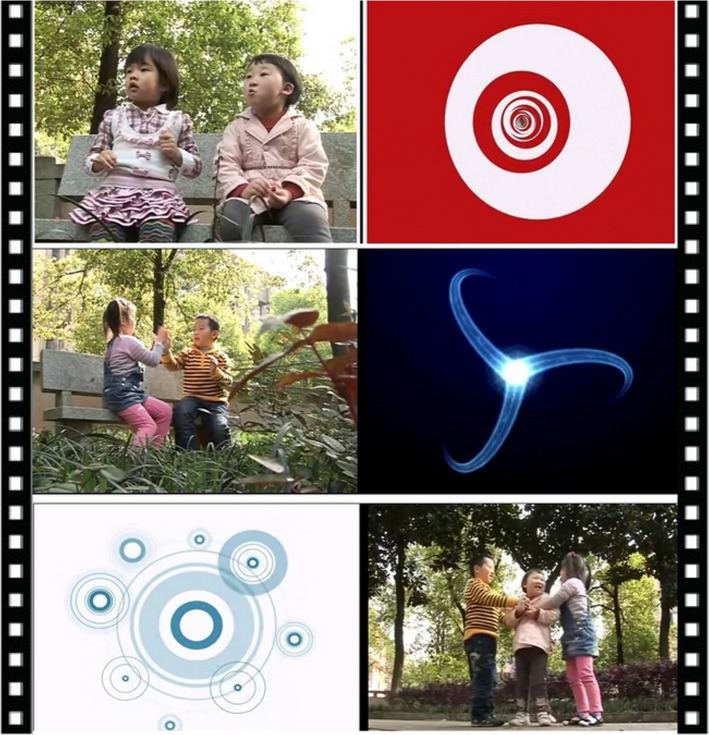
Sample stimuli illustrating three movie frames in Part II. The one-minute movie was recorded by the researchers with parental permission. The individuals in this figure have given written informed consent (as outlined in PLOS consent form) to publish these case details.

In Part II, modified stimuli were used in which the DGIs consisted of images of letters and geometric shapes, downloaded from the Internet. The DSIs were three short sequences of children playing games recorded by the researchers with parental permission. Each stimulus slide consisted of paired DSI and DGI displayed side-by-side on the screen at 20 s intervals in a random order (Fig. 1). Audio information was not presented. Prior to each sequence, an attention-grabbing animation (a penguin) appeared in the center of the screen to reorient attention and to ensure that all scanning patterns began at a position equidistant from the paired DSI and DGI stimuli.

### Eye-tracking procedure

Tests were carried out in a single session in a research laboratory located in the Second Xiangya Hospital, Central South University. Children were seated on a parent’s lap or in a chair, approximately 70 cm from a 60-cm widescreen computer monitor. The lights were off during testing and a partition separated the operator from the children. To obtain calibration information, children were first shown images of an animated penguin that appeared in one of nine locations on the screen. If calibration quality (displayed automatically as good, poor or fair on the computer monitor) was poor or fair for any of these points, the calibration process was repeated. Prior to the test, children were told that some movies were going to be shown on the computer screen and that they were free to watch them if they so wished.

### Data analysis

In this study, the areas of interest (AOIs) were located in two equal-sized rectangles, which contained DSIs and DGIs adjacent on the screen. Using Data View, the built-in data analysis software of Eyelink 1000, all fixation data were abstracted. Fixation duration, number of fixations, gaze duration and time of first fixation were analyzed. Measurement data in which fixations fell outside the AOI or the total viewing time was less than 30 s were excluded from the analysis. Fixation duration was calculated as the duration of fixation on AOIs divided by the duration of the stimulus display and then multiplied by 100.

Because there were no significant differences between groups for chronological age and sex, these variables were not considered in subsequent analyses. Repeated measures analysis of variance (ANOVA) was performed to compare the fixation duration, the percentage of fixation duration, the number of fixations, the gaze duration and the time of first fixation within the DSIs or DGIs between the two groups. In this model, the within-subject factor was experimental classification (Part I vs. Part II) and the between-subject factor was group (ASD vs. TDC), with PPVT VIQ used as a covariate. Effect sizes (partial eta—squared, *ηp2*, for F statistics) are reported together with *p*-values for significant main effects, with *ηp2* values above 0.01, 0.06 and 0.14 typically considered to reflect a small, medium and large effects, respectively. If significant interaction effects were found, one-way ANOVA tests were conducted and Bonferroni-corrected *p*-values were used. To investigate the capacity of this approach to discriminate between children with an ASD and TDC, a receiver operating characteristic (ROC) curve was generated.

To investigate the spatial distribution of fixations in the AOI, iMap [20], which is a data-driven approach, was used to produce statistical fixation maps of the eye-movement raw point-of-regard data generated in this study. Using iMap, based on a method used for functional magnetic resonance imaging analysis (fMRI), fixation point distribution was smoothed by convolving Gaussian kernels, then aligned, translated and overlaid. Furthermore, pixel-wise multiple comparison analyses were processed, and maps of areas exceeding the probability level were calculated according to random field theory. Compared to the traditional AOI analysis, iMap appears to be more objective, accurate and visual and to provide a better spatial resolution, through which differences between distinct groups or conditions in spatial distribution of fixation points can be detected.

## Results

### Fixation duration and the percentage of fixation duration

**Fig 2:**
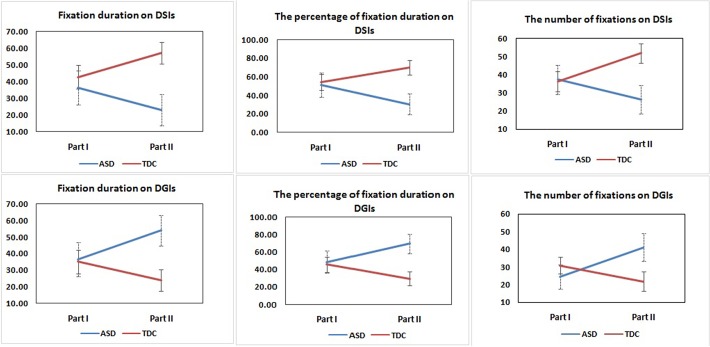
Estimated marginal means for fixation duration, the percentage of fixation duration and the number of fixations on DSIs and DGIs by the ASD and TDC groups in Part I and Part II. PPVT VIQ is co-varied.

There were no statistical differences in total fixation duration between the ASD and TDC groups in the two parts of the experiment [Part I: t (31) = 0.588 *p* = 0.561 Part II: t (14.071) = 1.310 *p* = 0.211] (Table 2). Analysis of the fixation duration on DSIs and DGIs measured during Parts I and II revealed no significant main effect for experimental classification [DSIs: F (1, 30) = 0.534 *p* = 0.470 *ηp2* = 0.017; DGIs: F (1, 30) = 0.509 *p* = 0.481 *ηp2* = 0.017] and group [DSIs: F (1, 30) = 2.224 *p* = 0.146 *ηp2* = 0.069; DGIs: F (1, 30) = 1.358 *p* = 0.253 *ηp2* = 0.043]. A slightly significant interaction was detected between group and experimental classification in fixation duration on DSIs [F (1, 30) = 3.797 *p* = 0.061 *ηp2* = 0.112] and DGIs [F (1, 30) = 4.037 *p* = 0.054 *ηp2* = 0.119] (Fig. 2). One-way ANOVA indicated that, in Part I of the study, there was no statistical difference in the fixation duration on DSIs or DGIs between the two groups. But in Part II, significant differences were found in fixation duration on DSIs [F (1, 31) = 19.247 *p* < 0.001] and DGIs [F (1, 31) = 16.016 *p* < 0.001] (This met the Bonferroni-correction threshold of ˂0.025) (Table 2).

**Table 2:** The total fixation duration, percentages of fixation duration and number of fixations, gaze duration and the time of first fixation for children with ASD and TDC.

		ASD (n = 13)	TDC(n = 20)	*F*	*p-value*
**Part I**	**Total fixation duration, mean (SD)**	75.17(8.68)	76.58(5.13)	t = 0.588	0.561
	**Fixation duration on DSIs, mean (SD)**	36.16(21.55)	42.95(14.97)	1.147	0.292
	**Fixation duration on DGIs, mean (SD)**	39.01(20.93)	33.62(16.01)	0.700	0.409
	**Percentage of fixation duration for DSIs (%)**	47.91(26.57)	56.46(19.83)	1.121	0.298
	**Percentage of fixation duration for DGIs (%)**	52.09(26.57)	43.54(19.83)	1.121	0.298
	**Number of fixations for DSIs, mean (SD)**	30.23(13.85)	40.85(14.86)	4.238	0.048
	**Number of fixations for DGIs, mean (SD)**	24.38(12.31)	30.90(11.91)	2.296	0.140
	**Gaze duration to DSIs, mean (SD), (s)**	2.60(4.37)	0.99(0.66)	2.689	0.111
	**Gaze duration to DGIs, mean (SD), (s)**	1.16(1.04)	0.65(0.49)	3.494	0.071
	**Time of first fixation to DSIs, mean (SD), (s)**	0.75(1.15)	0.52(0.38)	0.684	0.415
	**Time of first fixation to DGIs, mean (SD), (s)**	0.45(0.34)	0.34(0.20)	1.195	0.283
**Part II**	**Total fixation duration, mean (SD)**	77.82(6.72)	80.36(2.44)	t = 1.310	0.211
	**Fixation duration on DSIs, mean (SD)**	27.98(15.23)	53.74(17.23)	19.247	<0.001*
	**Fixation duration on DGIs, mean (SD)**	49.84(15.27)	26.62(16.90)	16.016	<0.001*
	**Percentage of fixation duration for DSIs (%)**	35.86(18.59)	66.82(20.94)	18.763	<0.001*
	**Percentage of fixation duration for DGIs (%)**	64.14(18.59)	33.18(20.94)	18.763	<0.001*
	**Number of fixations for DSIs, mean (SD)**	30.23(12.13)	49.25(15.00)	14.632	0.001*
	**Number of fixations for DGIs, mean (SD)**	38.77(15.95)	23.30(12.23)	9.915	0.004*
	**Gaze duration to DSIs, mean (SD), (s)**	3.22(4.34)	1.40(1.83)	2.778	0.106
	**Gaze duration to DGIs, mean (SD), (s)**	1.67(4.33)	0.53(0.51)	1.368	0.251
	**Time of first fixation to DSIs, mean (SD), (s)**	0.73(0.83)	0.45(0.37)	1.759	0.194
	**Time of first fixation to DGIs, mean (SD), (s)**	0.33(0.20)	0.40(0.19)	1.129	0.296

ASD: autism spectrum disorder, TDC: typically developing children. The variables (except the total fixation duration in Parts I and II), were tested using one-way ANOVA.

*p < 0.025.

**Fig 3:**
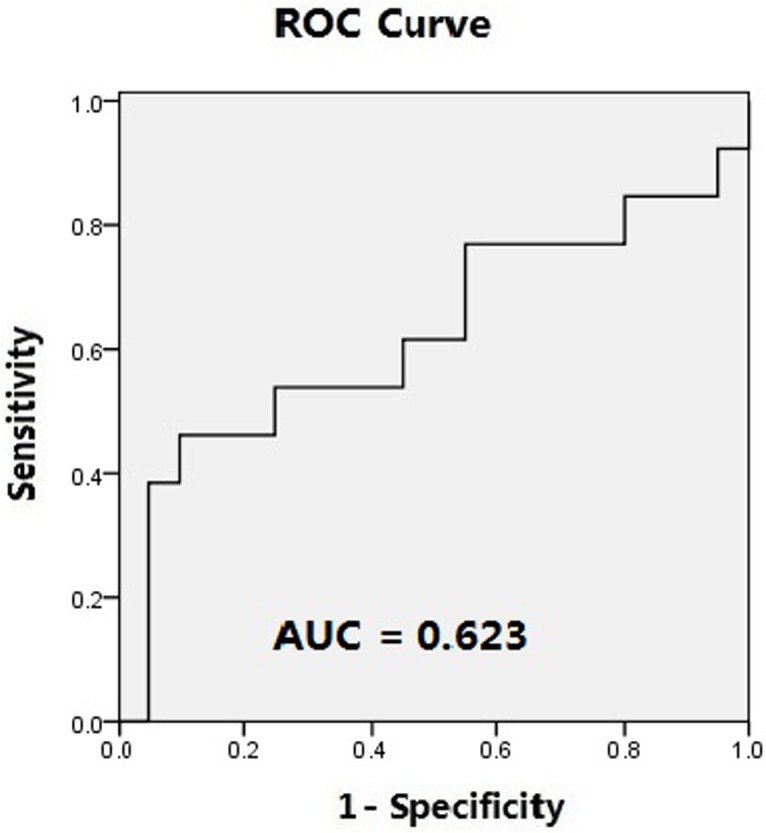
Receiver operating characteristic (ROC) curve demonstrating the sensitivity and specificity of Part I for identifying children with ASDs and typically developing children. AUC: area under the curve.

**Fig 4:**
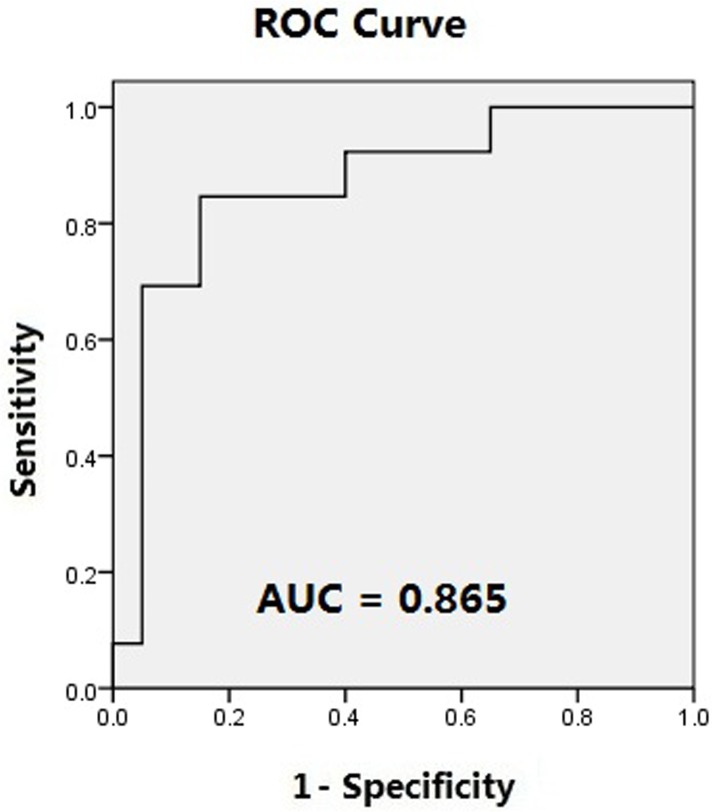
Receiver operating characteristic (ROC) curve demonstrating the sensitivity and specificity of Part II for identifying children with ASDs and typically developing children. AUC: area under the curve.

A significant interaction was detected between group and experimental classification in the percentage of fixation duration on DSIs and DGIs [DSIs and DGIs, F (1, 30) = 4.373 *p* = 0.045 *ηp2* = 0.127] (Fig. 2). One-way ANOVA indicated that, in Part I of the study, there was no significant difference in the percentage of fixation duration that children spent viewing the DSI or DGI stimuli between the ASD and TDC groups. However, in Part II, there was a significant difference between the percentage of fixation duration that children spent viewing the DSI or DGI stimuli between the ASD and TDC groups [F (1, 31) = 18.763 *p* < 0.001] (Table 2). ROC curve analysis indicated poor capacity of Pierce’s stimuli to distinguish between the ASD and TDC groups (area under the curve (AUC) = 0.623 ± 0.109; *p* = 0.238; Fig. 3), but a good capacity of the more complex stimuli to distinguish between the ASD and TDC groups (AUC = 0.865 ± 0.068; *p <* 0.001; Fig. 4). Eighty-five percent of participants in TDC group spent more than half of the viewing time fixated on the DSI stimulus in contrast to only 15.4% of participants in ASD group in Part II.

### Number of fixations

Analysis of the number of fixations on DSIs and DGIs measured during Part I and Part II revealed no significant main effect for group [DSIs: F (1, 30) = 1.172 *p* = 0.288 *ηp2* = 0.038; DGIs: F (1, 30) = 0.384 *p* = 0.540 *ηp2* = 0.013]. A significant main effect for experimental classification was detected in the number of fixations on DSIs [F (1, 30) = 4.230 *p* = 0.049 *ηp2* = 0.124], but not in the number of fixations on DGIs [F (1, 30) = 0.020 *p* = 0.888 *ηp2* = 0.001]. Significant interactions were detected between group and experimental classification in the number of fixations on DSIs [F (1, 30) = 5.737 *p* = 0.023 *ηp2* = 0.161] and DGIs [F (1, 30) = 6.093 *p* = 0.019 *ηp2* = 0.169] (Fig. 2). One-way ANOVA indicated that, in Part I, the significance level for the number of fixations on DSIs between the two groups was [F (1, 31) = 4.238 *p* = 0.048]; however, this did not meet the Bonferroni-correction threshold of less than 0.025. The significance level for the number of fixations on DGIs between two groups was *p* = 0.140. In Part II, there were significant differences in the number of fixations while viewing DSIs [F (1, 31) = 14.632 *p* = 0.001] and DGIs [F (1, 31) = 9.915 *p* = 0.004] between the two groups (Table 2).

### Gaze duration

Analysis of the gaze duration for DSIs and DGIs measured during Part I and Part II revealed no significant main effect for experimental classification [DSIs: F (1, 30) = 0.325 *p* = 0.573 *ηp2* = 0.011; DGIs: F (1, 30) = 0.009 *p* = 0.924 *ηp2* < 0.001] and group [DSIs: F (1, 30) = 1.523 *p* = 0.227 *ηp2* = 0.048; DGIs: F (1, 30) = 0.447 *p* = 0.509 *ηp2* = 0.015]. There was also no significant main effect for group and experimental classification interaction in the gaze duration for the DSIs [F (1, 30) = 0.472 *p* = 0.498 *ηp2* = 0.015] and the DGIs [F (1, 30) = 0.213 *p* = 0.648 *ηp2* = 0.007].

### The time of first fixation

Analysis of the time of first fixation for DSIs and DGIs measured during Parts I and II revealed no significant main effect for experimental classification [DSIs: F (1, 30) = 0.188 *p* = 0.668 *ηp2* = 0.006; DGIs: F (1, 30) = 0.069 *p* = 0.794 *ηp2* = 0.002] and group [DSIs: F (1, 30) = 0.497 *p* = 0.486 *ηp2* = 0.016; DGIs: F (1, 30) = 1.030 *p* = 0.318 *ηp2* = 0.033]. There was also no significant main effect for group and experimental classification interaction in the time of first fixation for the DSIs [F (1, 30) = 0.185 *p* = 0.670 *ηp2* = 0.006] and the DGIs [F (1, 30) = 0.844 *p* = 0.365 *ηp2* = 0.027].

### Fixation point distribution

**Fig 5:**
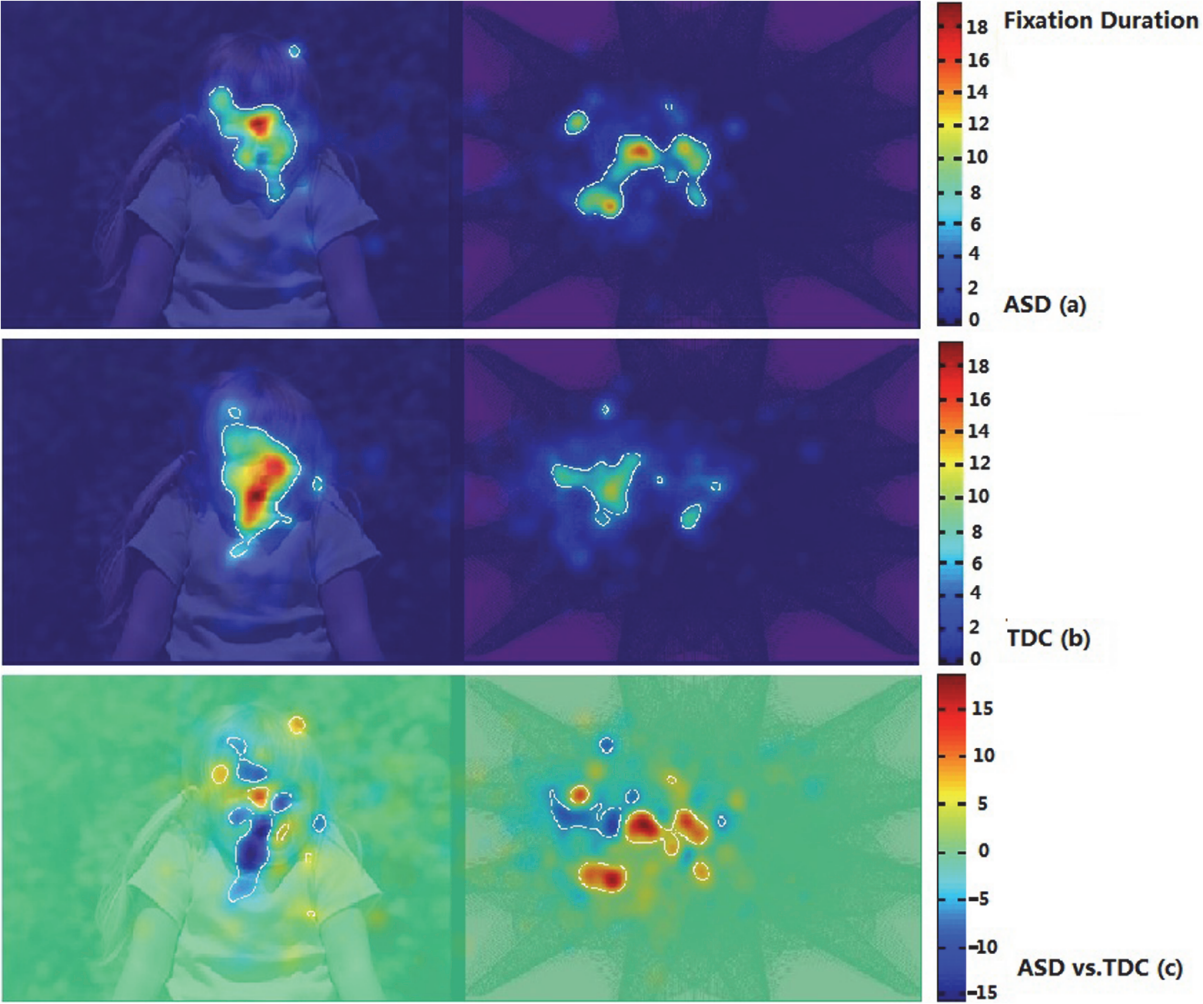
iMaps showing the distribution of fixation duration in the ASD group and the TDC group in Part I. ASD (a): Z-score maps showing the distribution of fixation duration in the ASD group; TDC (b): Z-score maps showing the distribution of fixation duration in the TDC group; Warm colors denote longer fixation duration and cold colors denote shorter fixation duration. ASD vs. TDC (c): Z-score difference map prepared by subtracting the fixation duration data of the TDC group from that of the ASD group. Red areas indicate positive results; the ASD group spent more time in these areas than did the TDC group. Blue areas represent negative results; the control group spent more time in these areas than did the ASD group.

**Fig 6:**
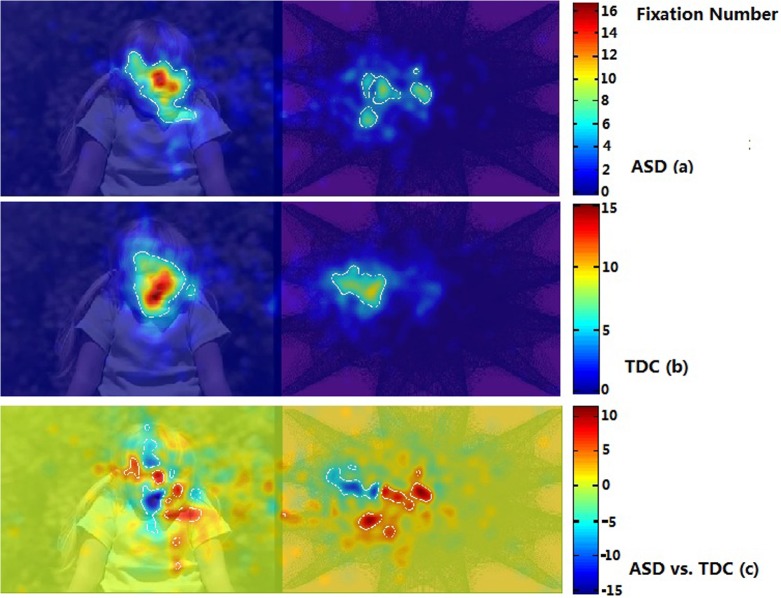
iMaps showing the distribution of the number of fixations in the ASD group and the TDC group in Part I. ASD (a): Z-score maps showing the distribution of the number of fixations in the ASD group; TDC (b): Z-score maps showing the distribution of the number of fixations in the TDC group; Warm colors denote greater numbers of fixations and cold colors denote fewer fixations. ASD vs. TDC (c): Z-score difference map prepared by subtracting the number of fixations in the TDC group from that in the ASD group. Red areas indicate positive results; the ASD group exhibited a greater fixation number in these areas than did the TDC group. Blue areas represent negative results; the TDC group exhibited a greater fixation number in these areas than did the ASD group.

**Fig 7:**
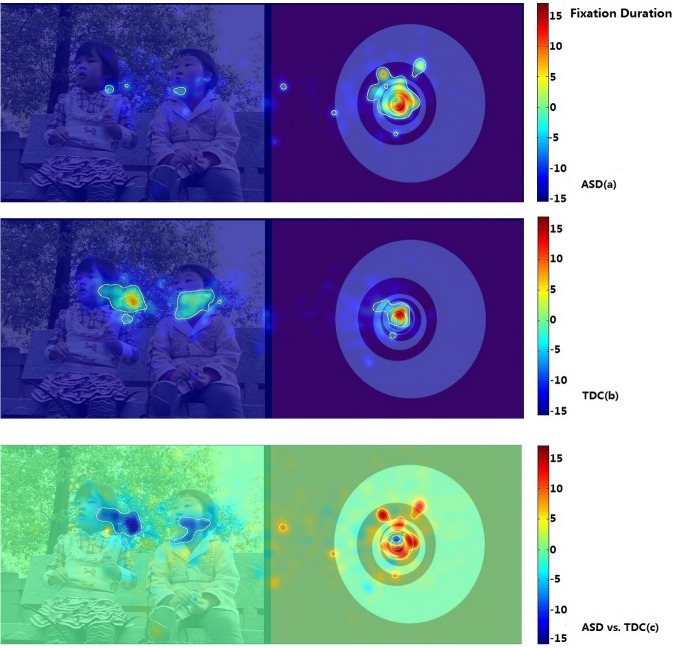
iMaps showing the distribution of fixation duration in the ASD group and the TDC group in Part II. ASD (a): Z-score maps showing the distribution of fixation duration in the ASD group; TDC (b): Z-score maps showing the distribution of fixation duration in the TDC group; Warm colors denote longer fixation duration and cold colors denote shorter fixation duration. ASD vs. TDC (c): Z-score difference map prepared by subtracting the fixation duration data of the TDC group from that of the ASD group. Red areas indicate positive results; the ASD group spent more time in these areas than did the TDC group. Blue areas represent negative results; the TDC group spent more time in these areas than did the ASD group.

**Fig 8:**
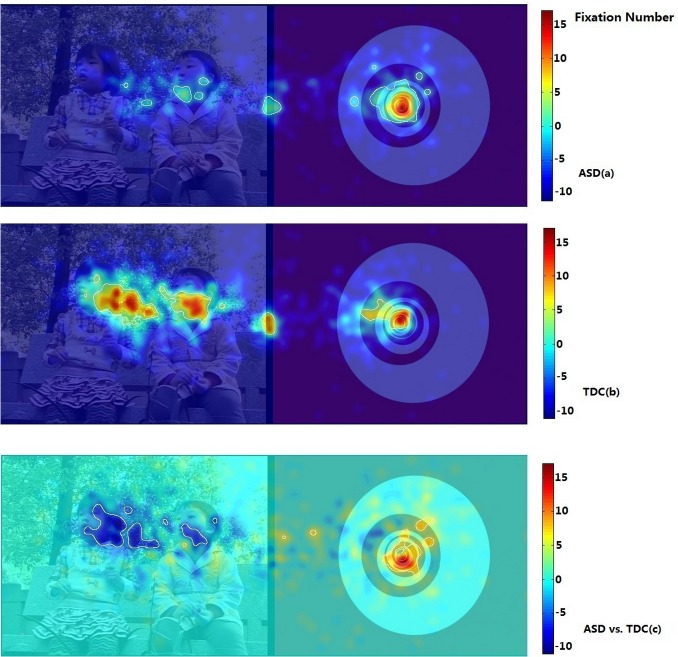
iMaps showing the distribution of the number of fixations in the ASD group and the TDC group in Part II. ASD (a): Z-score maps showing the distribution of the number of fixations in the ASD group; TDC (b): Z-score maps showing the distribution of the number of fixations in the TDC group; Warm colors denote a greater number of fixations and cold colors denote fewer fixations. ASD vs. TDC (c): Z-score difference map prepared by subtracting the number of fixations in the TDC group from that in the ASD group. Red areas indicate positive results; the ASD group spent more fixation number in these areas than did the TDC group. Blue areas represent negative results; the TDC group spent more fixation number in these areas than did the ASD group.

Using iMap, we compared the duration and number of fixations in the ASD and TDC groups. The Z-score maps of fixation duration and number of fixations in Part I are presented in Figs. 5 and 6. The Z-score maps of fixation duration and number of fixations in Part II are presented in Figs. 7 and 8. Areas with significantly greater (or lesser) number of fixations or fixation duration between the two groups were plotted with white contour lines (all *P* < 0.05). In the difference maps, red areas indicate positive results; the ASD group spent more time (greater number of fixations) in these areas than did the TDC group. Blue areas represent negative results; the control group spent more time (greater number of fixations) in these areas than did the ASD group.

In Part I, no significant difference in the distribution patterns of fixation duration was found between the two groups (Fig. 5A and 5B). However, the difference map showed that the ASD group spent less time on DSIs showing a face (from the observer’s perspective) (Fig. 5C). Both the ASD and TDC groups had a greater number of fixations on the DSIs than on the DGIs (Fig. 6A and 6B). However, the difference map showed a difference in the spatial distribution of the number of fixations within DSIs and DGIs between the ASD group and the TDC group (from the observer’s perspective) (Fig. 6C).

Part II clearly showed that, when presented with DGIs and DSIs, children with ASDs spent most of their fixation time on DGIs (Fig. 7A), while TDC spent an equal amount of time viewing both types of images (Fig. 7B). The difference map showed that the time spent viewing DGIs was significantly greater among children with ASDs than among TDC, while the time viewing DSIs was significantly less among the children with ASDs (Fig. 7C). In addition, among children with ASDs, most fixations were distributed within the DGIs (Fig. 8A), while TDC had an equal number of fixations among the DSIs and DGIs (Fig. 8B). Fig. 8C shows that the number of fixations distributed among the DGIs was significantly greater among children with ASDs than among TDC, whereas the number of fixations distributed among the DSIs was greater among TDC than among children with ASDs.

## Discussion

In this study, we aimed to explore the visual social attention patterns of children aged 4 to 6 years with ASDs and to determine whether the findings of Pierce et al. hold true for older children. Furthermore, we aimed to determine whether more complex visual social stimuli can be used to differentiate children with ASDs from TDC. Our results suggest that the stimuli used in the study conducted by Pierce et al. are not suitable for identifying ASDs in children aged 4 to 6 years. As expected, the more complex visual social stimuli were shown to elicit differences in visual attention preference patterns between children with ASD and TDC.

The AOI and iMap analyses of Part I of this study indicated similar visual attention preference pattern in children with ASD and TDC. This finding is inconsistent with the results of the study conducted by Pierce et al., which showed that individuals with ASD spent less time on the DSIs than the controls[9]. Since the effect size was small, one possibility for these negative findings may be the small sample size. Another possibility for these negative findings may be the sample age and the intervention of the children with ASD. Children with ASD, once diagnosed, are likely to receive various types of treatments in which trainers and parents teach them to pay more attention to humans; thus, their visual preference will change to a certain extent. However, additional novel findings were revealed by the data-driven approach used in the present study. Relative to the TDC group, the fixation duration on the part of the DSIs showing a face was shorter in the ASD group. This finding is consistent with results from previous studies showing that individuals with ASDs were less focused on the face than controls [21–23]. For example, Hosozawa et al.[24] found that children with ASD looked away from actors prematurely during speech episodes and looked less at faces in general compared to children with specific language disorders and TDC. Chawarska et al. [25] noted that, regardless of the context, 6 month-old infants who subsequently receive a diagnosis of ASD during the third year of life demonstrated decreased attention to social scenes and to faces while watching a continuous video clip depicting an actress engaged in different activities associated with varied social content.

Our results in Part II of the study (AOI and iMap analysis) revealed that the TDC group showed a preference for DSIs, while the ASDs preferred DGIs. The DSIs of Part II depicted children playing rather than one child doing yoga, as was the case in Part I. It is well established that play is a reflection of children’s social competence with peers[26], with older children exhibiting an increased preference for socially organized play[27]. Parten found that solitary play was most common at 18 months to 2 years but that there was a distinct decline in the importance of solitary play at 3 to 4 years of age. Onlookers were most prevalent among the 2.5- to 3-year-old age group. Parallel play groups were observed most often among the 2-year-olds, and least as often among children aged three to four years. Children’s preference for associative group play increased with age, and was most frequent in the 4- to 4.5-year old group on their study. There is a marked increase in organized supplementary play beginning during the third year. It has been proposed that parallel, associative and cooperative or organized supplementary play represent positive indices of social participation[27]. Our results indicated that TDC preferred the DSIs in Part II compared with the DSIs in Part I. However, the children in the ASD group did not appear to have developed this preference, and were more focused on the DGIs in Part II than the DGIs in Part I. This is also consistent with the idea that the AOIs of TDC extend with age, and that these children explore more complex stimuli, both social and non-social. However, children with autism reduce their visual social attention to more complex social stimuli as they grow up [13].

The differences noted between the results obtained in Part I and Part II of the present study suggest that the nature of visual social stimuli influence the results. We speculate that the DSIs shown in Part I, which include only one person moving, are only suitable for identifying toddlers with ASDs, while DSIs used in Part II, which depict more complex social interaction information are more suitable for identifying ASDs in older children. Previous studies have indicated a relationship between the stimuli used and the results obtained. For example, in a study that aimed to determine how children with ASDs process visualization of the human face, the researchers suggested that the face-processing deficit associated with autism appears to be at least partially dependent on stimuli being both realistic and social in nature[28]. Furthermore, Sasson et al.[7] showed that children with ASDs presented with the human face and objects not associated with restricted interest side-by-side showed similar fixation patterns as did TDC. In contrast, when the stimuli used depicted the human face and objects associated with restricted interests, the fixation patterns of the children with ASDs were different from those of TDC. Chawarska et al.[14]also reported that the abnormality in the fixation patterns of children with ASDs toward stimuli showing social information was associated with the scene included in the visual stimuli. It can be speculated that children with ASDs are better able to process the social interactions portrayed in the stimuli involving a single individual compared to stimuli showing multiple interacting individuals in a dynamic scene.

It is also worth noting that the gaze duration and the time of the first fixation on the DSIs and DGIs did not differ between the two groups in Parts I and II. These measures indicate the stimuli that received attentional priority in the scene, rather than simply the stimuli that received the greatest amount of viewing time. The results in the present study indicated that the attentional priority for DSIs was generally similar between children with ASD and the TDC. This is inconsistent with a previous study that suggested that the non-ASD group showed a greater bias for first fixations on the person presented in the scene than did the ASD group[29]. The heterogeneity in sample age and the social stimuli content may be important factors responsible for this discrepancy.

Several limitations of this study should be mentioned. First, although all of the subjects in the current study were Chinese children, the video shown in Part I depicted mainly Caucasian children; therefore, racial biases need to be considered when generalizing the conclusions relating to this part of the study. Previous studies have provided evidence for the effect of race on processing. For example, there are studies showing that the duration of fixation of children looking at faces of their own race is extended relative to that when observing other races [30,31]. Furthermore, it has been shown that children are subject to more errors and difficulties in encoding cross-race faces [32]. Therefore, further studies are needed to investigate the impact of racial biases on the results of our study. Second, the sample size used here is relatively small and a study conducted in a larger sample is required to validate our results. Third, our sample included only children aged 4 to 6 years and further longitudinal studies are required to explore the changes in the visual preference pattern in ASDs systematically. Also, an assessment of visual functions among the children was not performed to exclude visual impairments and we based our information on parental reports. Finally, general cultural differences between Caucasian and Chinese children should be considered in the interpretation of these visual attention data.

In conclusion, the findings of this study suggest that social stimuli comprising only one person cannot be used to detect differences between children with ASD and TDC in the 4- to 6-year-old age group. When using social stimuli that showed two or more children playing, children with ASDs exhibited a preference for DGIs, while the fixation time of TDC on DSIs was greater. Among children aged 4 to 6 years with ASDs, visual attention preference patterns are modulated by the social characteristics of the stimuli to a certain degree. These findings provide the basis for further examination of the influence of social stimuli on visual attention preference patterns among individuals with ASD by varying the number of people displayed as well as the context in which the people appears (e.g., two or more people interacting vs. not interacting with each other). Future investigations of the nature of social attention impairments associated with autism should consider the cultural nature of the stimuli used in the investigation.

## Supporting Information

S1 Dataset The Dataset underlying the findings reported in this study. P_: Part I; S_: Part II.
(XLS)
